# Estrogen receptor α/prolactin receptor bilateral crosstalk promotes bromocriptine resistance in prolactinomas

**DOI:** 10.7150/ijms.51176

**Published:** 2020-10-23

**Authors:** Zhengzheng Xiao, Xiaoli Yang, Kun Zhang, Zebin Liu, Zheng Shao, Chaojun Song, Xiaobin Wang, Zhengwei Li

**Affiliations:** 1 Department of Henan Key Laboratory of Cancer Epigenetics; Cancer Institute, Department of Neurosurgery, The First Affiliated Hospital and College of Clinical Medicine of Henan University of Science and Technology, Luoyang, Henan 471003.; 2 Department of General Practice, The First Affiliated Hospital and College of Clinical Medicine of Henan University of Science and Technology, Luoyang, Henan 471003.; 3 Spine Tumor Center, Department of Orthopedic Oncology, Changzheng Hospital, Second Military Medical University, Shanghai 210011.; 4 Carson International Cancer Centre, Shenzhen University General Hospital and Shenzhen University Clinical Medical Academy Centre, Shenzhen University, Shenzhen, Guangdong 518000.; 5 Department of Neurosurgery, Zhongnan hospital of Wuhan university, Wuhan, Hubei 430071, P.R. China.

**Keywords:** estrogen receptor α, prolactin receptor, bromocriptine, prolactinoma

## Abstract

Prolactinomas are the most common type of functional pituitary adenoma. Although bromocriptine is the preferred first line treatment for prolactinoma, resistance frequently occurs, posing a prominent clinical challenge. Both the prolactin receptor (PRLR) and estrogen receptor α (ERα) serve critical roles in the development and progression of prolactinomas, and whether this interaction between PRLR and ERα contributes to bromocriptine resistance remains to be clarified. In the present study, increased levels of ERα and PRLR protein expression were detected in bromocriptine-resistant prolactinomas and MMQ cells. Prolactin (PRL) and estradiol (E2) were found to exert synergistic effects on prolactinoma cell proliferation. Furthermore, PRL induced the phosphorylation of ERα via the JAK2-PI3K/Akt-MEK/ERK pathway, while estrogen promoted PRLR upregulation via pERα. ERα inhibition abolished E2-induced PRLR upregulation and PRL-induced ERα phosphorylation, and fulvestrant, an ERα inhibitor, restored pituitary adenoma cell sensitivity to bromocriptine by activating JNK-MEK/ERK-p38 MAPK signaling and cyclin D1 downregulation. Collectively, these data suggest that the interaction between the estrogen/ERα and PRL/PRLR pathways may contribute to bromocriptine resistance, and therefore, that combination treatment with fulvestrant and bromocriptine (as opposed to either drug alone) may exert potent antitumor effects on bromocriptine-resistant prolactinomas.

## Introduction

Pituitary adenomas are frequently occurring primary brain tumors that account for 15-20% of all intracranial neoplasms 1-3. Prolactinomas are the most predominant type of functional pituitary adenoma, constituting ~40% of total pituitary adenomas globally 4,5. Over the past decades, there have been substantial breakthroughs in the understanding and management of prolactinomas; bromocriptine is the first line treatment for prolactinomas, yet 30% of patients ultimately develop resistance 3,6,7. These clinical phenomena highlight an urgent requirement for alternative treatment strategies.

Findings from our clinical practice indicate that 23% of pregnant women possess larger prolactinomas, and the use of estrogen-based contraceptives is an indicated risk factor for prolactinoma development 8; these findings suggest that estrogen may contribute to the progression of prolactinomas. As estrogen has been reported to regulate the growth, differentiation and function of various tissue types via estrogen receptor α (ERα), and as bromocriptine-resistant prolactinomas possess high expression levels of ERα 9-14, it was hypothesized that ERα signaling may be involved in the mediation of bromocriptine resistance.

In addition to estrogen, the hormone prolactin (PRL) also promotes carcinogenesis through the PRL receptor (PRLR)-dependent pathway. PRLR has been detected in the majority of prolactinomas, and has also been associated with prolactinoma size and invasiveness 15-18. Since previous studies have suggested that the interaction between local hormones exacerbates drug resistance 19, it was hypothesized that crosstalk between estrogen/ERα and PRL/PRLR signaling may result in bromocriptine resistance.

To elucidate the effects of the ERα/PRLR signaling interaction on bromocriptine resistance in prolactinomas, the present study aimed to establish a bromocriptine-resistant prolactinoma MMQ cell line (MMQ/BRO), which was employed alongside primary cultured human pituitary adenoma (HPA) cells. Loss-of-function experiments were then performed using an shRNA kit and the ERα antagonist fulvestrant; To the best of our knowledge, the present study demonstrates for the first time that estrogen upregulates PRLR expression via pERα, while in turn, PRL induces ERα phosphorylation in prolactinomas. Thus, the estrogen/ERα and PRL/PRLR signaling pathways form a reciprocal positive regulatory loop that promotes bromocriptine resistance by activating JNK-MEK/ERK-p38 signaling and cyclin D1 downregulation. Moreover, when used as an adjuvant therapeutic, fulvestrant can restore bromocriptine sensitivity in prolactinomas. Clinically, it may be crucial to block this crosstalk to achieve optimal therapeutic activity. Currently, as cabergoline has not been approved for clinical application in China yet, this finding is of translational significance in the implementation of bromocriptine-associated prolactinoma resistance therapy.

## Materials and Methods

### Reagents and antibodies

PRL, estradiol (E2), fulvestrant, the JAK2 inhibitor AG-490, the PI3K inhibitor Wortmannin, the ERK inhibitor U0126, and the STAT5 inhibitor pimozide were purchased from Selleck Chemicals. Anti-dopamine D2 receptor (D2R; cat. no. ab85367), ERα (E115; cat. no ab32063), CD133 (cat. no. ab16518) and Ki67 (cat. no. ab16667) antibodies were obtained from Abcam. PRLR (cat. no. #13552), p-ERα (Ser118; cat. no. #2511), Stat5 (cat. no. #94205), p-Stat5 (Tyr694; cat. no. #9359), AKT (cat. no. #4691), p-Akt (Ser473; cat. no #4060) and p-JAK2 (Tyr1007/1008; cat. no. #3776) primary antibodies were purchased from Cell Signaling Technology. Inc. Erk2 (sc-1647), p-ERK (cat. no sc-7383), JAK2 (cat. no. sc-390539), cyclin D1 (cat. no. sc-8396), P38 (cat. no. sc-81621) and β-actin (cat. no. sc-81178) primary antibodies were purchased from Santa Cruz Biotechnology, Inc, horseradish peroxidase (HRP)-conjugated Goat anti-Mouse IgG (Cat#G-21040), Goat anti-Rabbit IgG (Cat#G-21234) secondary antibodies were purchased from Thermo Fisher SCIENTIFIC.

### Cell culture

The MMQ rat prolactinoma cell line was purchased from the Cell Culture Centre of the Institute of Basic Medical Sciences, Chinese Academy of Medical Sciences (Beijing, China). Bromocriptine-resistant cells (MMQ/BRO) were established over 6 months by exposing MMQ cells to increasing concentrations of bromocriptine (5-100 µM), combined with intermittent high-dose pulses (25, 50, 75 and 100 µM). MMQ/BRO cells were subsequently cultured with 15 µM bromocriptine to preserve resistance. All cells were maintained in F12 culture medium (Wuhan Boster Biotechnology Co., Ltd.) supplemented with 5% fetal bovine serum (FBS; Wuhan Boster Biotechnology Co., Ltd.), 10% horse serum (Wuhan Boster Biotechnology Co., Ltd.), penicillin and streptomycin (100 μg/ml each; Genom Biotech Pvt., Ltd.) in a humidified incubator at 37 °C (5% CO_2_).

### Primary culture of pituitary adenoma tissues

Human pituitary adenoma tissues were processed directly following surgery, and according to the standard protocols described in our previous study 20. The Human Tumor Dissociation kit (Miltenyi Biotec. cat. no.130-095-929) was used with the gentleMACS™ Dissociator (Miltenyi Biotec. cat. no. DXT-130-096-730) to enzymatically digest the tissues, and human Anti-Fibroblast MicroBeads (Miltenyi Biotec. cat. no. 130-050-601) were used to filter the tissue lysates. For each experiment, tissues from one patient was used. In order to obtain enough prolactinoma tissues for primary culture, only huge and invasive prolactinoma tissues was selected. After surgery, tumor specimens were placed in ice and transfered to lab. Tumor specimens were placed in complete DMEM, supplemented with Penicillin 100U, Streptomycin 100 g/ml, 15 mM HEPES, 3151 mg/L Glucose, 55 mg/L sodium pyruvate, 365 mg/L L-Glutamine, 1200 mg/L Sodium bicarbonate. Ph 7.0-7.4 (Wuhan Boster Biotechnology Co., Ltd. cat. no. PYG0085). The pituitary tumor standard in a sterile culture dish, with sterile pre-cooled PBS clear-wash standard, wash off the surface of blood, with sterile crucible to tear off the necrotic tissue. Aseptic tweezers pick up the tissue. Dip the tissue in the DMEM medium, cut the tissue to 1mm with sterile scissors, until the tissue can be blowed with 1mL pipette. Then Human Tumor Dissociation kit was added, and put into a 37 °C carbon dioxide incubator 60min, every 10 minutes to remove the pipette blow 1 time. Using a pipette to absorb the fluid and filter the liquid through the aseptic filter in a 50mL centrifuge tube. Use DMEM culture base neutralize the Enzyme in the Dissociation kit in the fine cell suspension of 50 mL centrifugal tube. 800 r/min centrifugal for 3min (centrifugation radius 200mm). Discard on the Supernatant, the Precipitates was resuspended in a dish of a DMEM medium containing l0% bovine serum. To obtain adenoma cell preparations deprived of rapidly dividing fibroblasts, dispersed cells were filtered through a magnetic bead column coated with human Anti-Fibroblast MicroBeads (Miltenyi Biotec. cat. no. 130-050-601), according to the manufacturer's specifications. The resulting cells were plated in 24-well culture plates at a density of 10^5^ cells/well in 1 ml of the complete DMEM medium. Depending on the available tissue, 50×10^6^ isolated cells/adenoma were obtained. After 3 days, the culture medium was collected for hormone determination, 5 μM BRDU (B5002, Sigma, cat. no. 59143) was added into 1 ml DMEM to suppress the proliferation of any remaining contaminating fibroblasts.

### CD133+/nestin+ primary cultured human pituitary adenoma (HPA) cell isolation

CD133+/nestin+ HPA cells were isolated from human primary cultured pituitary adenoma cells using CD133 (Miltenyi Biotec, Cambridge, USA, cat. no. 130097049) and Anti-Nestin Magnetic Beads (Sinobiological, Beijing, China. cat. no. MB106970-T46) as previously described 20,21. The QuadroMACS™ Separation Unit (Miltenyi Biotec) was used for isolation. Prior to purification, the MACS® (25 LD columns, Miltenyi Biotec, Germany) columns were filled with warmed (37 °C) RPMI or PBS. When the purification was carried out in order to synchronize the culture, the experimentation was performed under sterile conditions. The cells were digested and scattered, cells were then deposited on the top of the column (typically, 1 mL at 25-50% haematocrit) which was held in a Quadro MACS® magnetic support. The column was removed from the magnetic support and a further 4 mL (dead volume of the column) of culture medium added and the eluent recovered. This eluent was then centrifuged (800 g, 3 min) and the supernatant was discarded. The pellet was resuspend and cultured. To prevent differentiation, the cells were cultured in serum-free tumor sphere medium, and plated in an ultra-low attachment plate. Cell culture mediem is DMEM/F12 medium (Wuhan Boster Biotechnology Co., Ltd. cat. no.PYG0004), N2-supplement (5 mL; Invitrogen, cat. no. 17502048), Penicillin 100u, Streptomycin 100 g/mL, 15 mm HEPES, 3151 mg/L Glucose, 55 mg/L sodium pyruvate, 365 mg/L L-Glutamine, 1200 mg/L Sodium bicarbonate. Ph 7.0-7.4. N-acetylcysteine (60 μg/mL; Sigma, cat. no. A0737), neural survival factor-1 (10 mL; Lonza, cat. no. CC-4323), epidermal growth factor (20 ng/mL; ROCHE, cat. no. DXT-11376454001), basic fibroblast growth factor (20 ng/mL;Shanghai YIJI, cat. no. HL669727), leukemic inhibitory factor (10 ng/mL; Sciencell, cat. no. DXT-123-07).

### Transfection and luciferase reporter assay

A human pPLK/GFP+Puro-ERα short hairpin (sh)RNA and pPLK/GFP+Puro-Prlr shRNA system was obtained from GeneAll Biotechnology Co., Ltd. cat. no. 5618). According to the protocols described in our previous study (13,21), 2×10^5^ HPA cells in DMEM/F12 medium were seeded into 6-well plates and cultured to ~70% confluence. The cells were then transfected with 100 nM control or sample shRNA using the Lipofectamine® 3000 (Thermo Fisher Scientific, Inc. Cat. no. L3000001) transfection reagent per the manufacturer's protocol. After 12 h, the medium was replaced with fresh medium. From the next day onward, the cells were selected using puromycin (2 mg/ml) for at least 3-4 days. Luciferase reporter assays were performed 48h after the transfections using the Dual-Luciferase Reporter Assay System (Promega Corporation). Cells were lysed with 400 μl Reporter Lysis Buffer, 200 μl *Renilla* Luciferase Assay Lysis Buffer or 400 μl Passive Lysis Buffer, then vortexed and centrifuged to remove debris. Luciferase activity was normalized to that of the *Renilla* luciferase internal control, and all experiments were performed in triplicate.

### Cell viability assay

The Cell Counting Kit 8 (CCK-8; Boster,china. cat. no. AR1199) was used to assess cellular proliferation, according to the manufacturer's protocol. The cells were cultured in phenol red-free DMEM/F12 medium supplemented with 1% charcoal-stripped FBS without estrogen for 24 h, and then seeded into 96-well plates (1×10^3^ cells/well) for overnight culture. The cells were treated with various concentrations of PRL or E2 in phenol red-free DMEM/F12 medium and cultured for 3 days; 10 μl CCK-8 was then added and the cells were incubated at 37 °C for a further 4 h. Colorimetric absorbance was measured at 450 nm using an ELISA microplate reader, with six replicates per experimental sample. The half maximal inhibitory concentration (IC_50_) was calculated using GraphPad Prism 8 (GraphPad Software, Inc.) and the cell viability rate was calculated as follows: Cell viability rate = [(OD of treated cells - OD of blank)/(OD of control cells - OD of blank)] × 100%.

### Western blot analysis

Prolactinoma specimens and cells were lysed in lysis buffer for 30 min on ice. The total protein was quantified using a Protein Assay kit (Boster, Inc. cat. no. AR0106). Equal amounts of protein (30 µg) were separated on 10-15% SDS-PAGE gels and transferred onto nitrocellulose membranes. After blocking with 5% milk in TBST for 2 h at room temperature, the membranes were incubated with the aforementioned primary antibodies (1:1,000) at 4 °C overnight. The membranes were then washed in TBST buffer and incubated with HRP-conjugated secondary antibodies (1:3000) for 2 h at room temperature. After a final wash step, the membranes where developed using SuperSignal West Pico ECL solution (Thermo Fisher Scientific, Inc. cat. no. 34580) and the protein bands were detected and visualized using the FluorChem™ E imaging system (ProteinSimple).

### Prolactinoma specimens

In the present study, primary specimens were obtained from 8 patients with prolactinoma who had received bromocriptine first-line treatment following relapse, and 8 control patients who responded positively to bromocriptine therapy, but who also received surgery for unrelieved vision loss or poor medical compliance. The diagnoses of clinical prolactinoma were according to clinical and hormonal evaluation, histological assessment and PRL exclusive immunoreactivity. The PRL blood levels of the patients ranged from 154 to 232 µg/L before receiving medication. In order to maximize the consistency of our tumor inclusion, we only included non-invasive prolactinoma patients who had been resistant from the beginning. Based on previous studies 4,22,23, bromocriptine resistance was defined as the failure to normalize PRL levels or to reduce tumour size by ≥50%, following ≥15 mg/day bromocriptine for ≥3 months. All patients (9 men and 7 women; age range, 16-63 years; mean age, 43.50±11.00 years) received surgery between July 2013 and July 2019 at the Department of Neurosurgery, the First Affiliated Hospital of Henan University of Science and Technology (HUST; Luoyang, China). There was no relation between the serum PRL levels, tumor size and the bromocriptine resistance. Each tissue sample was bisected; one half was frozen for protein and RNA extraction, and the other half was fixed and embedded in paraffin for histological and immunohistochemical analysis. Written informed consent was obtained from all patients and the study protocols were approved by the Research Ethics Committee of the HUST.

### Immunohistochemical staining

For immunohistochemistry, tissue sections were fixed with 10% Neutral buffered formalin overnight at RT followed by an embedding in paraffin wax. 4 µm sections were deparaffinized with xylene and then rehydrated with distilled water through an ethanol series. Antigen retrieval was conducted with steaming slides in 0.01 M sodium citrate buffer (pH 6.0) at 99-100 °C for 20 min, and endogenous peroxidases were blocked using 3% hydrogen peroxide in methanol at RT for 10 min. The slides were subsequently blocked with 10% normal horse serum (Sigma, H1270) in TBST at RT for 1 h followed by primary antibody incubation (CD133, 1:500; and Ki67, 1:1,000) at 4 °C overnight. The slides were then washed with TBST and incubated with biotinylated anti-mouse IgG secondary antibody (Vectastain Elite ABC Kit, Vector Laboratories, cat. no. PK-7200) for 30 minutes at room temperature, followed by incubation in Vectastain Elite ABC Reagent (Vectastain Elite ABC Kit, Vector Laboratories, cat. no. PK-6100) for 30 minutes at room temperature. For all slides, a DAB Substrate Kit (Vector Laboratories, cat. no. SK-4100) was used according to the manufacturer's instructions. The sections were then counterstained with Meyer's hematoxylin (Abcam, china. cat. no. ab220365) for 1 min at RT, dehydrated in series ethanol and xylene, and mounted with permount. Slides were analyzed using a light microscope (Nikon ECLIPSE E100 microscope, Japan) under 40X or 200X magnification and Nikon Elements Imaging System Software.

### RNA extraction and qPCR

For Real-time RT-PCR, total RNA was extracted using TRIzol reagent (Thermo Fisher Scientific. cat. no. 15596026), and 1 µg of RNA was used for the RT reaction with random primer (Thermo Fisher Scientific. cat. no. 48190011), dNTP mix (Thermo Fisher Scientific. cat. no.18427013), and M-MuLV Reverse Transcriptase (New England Biolabs. cat. no. M0253L). Quantification of mRNA was determined using a SYBRGreen supermix (Bio-Rad. cat. no. 1708880). The PCR conditions were as follows: 94 °C for 2 minutes, 40 cycles of 94 °C for 30 seconds, 56 °C for 30 seconds, and 72 °C for 30 seconds. The triplicate samples were amplified in 20 µL reactions with gene-specific primers. The triplicate samples were amplified in 20 µL reactions with gene-specific primers. The mRNA abundance for each gene of interest was normalized to that of GAPDH. The qPCR primers are as follows: PRLR: forward: 5'-GAGAAGGCAGCCAACATGAAG-3' reverse: 5'-TGAATGAAGGTCGCTGGA CTC-3'; ERα: forward: 5'-GAATGTGCCTGGC TAGAGATCCT-3' reverse: 5'-TTCCTGTCCAAGAGCA AGTTAGG-3'. GAPDH: forward: 5'-CCACTCC TCCACCTTTGACG-3' reverse: 5'-CCACCCAAATGTTG CTGTA G-3'.

### *In vivo* nude mouse model

As described in previous studies 20,24, Female nude mice (age, 4 weeks; weight, 16-18 g) were maintained in the experimental animal center of the HUST in a pathogen-free environment. Animal research was approved by the Institutional Animal Ethics Committee of the HUST. In order to increase the tumor formation rate, human prolactinoma tissue suspension in 100 μl PBS and Matrigel mix (1:1) was subcutaneously injected into three nude mice of one group. Following tumor formation, the mice were sacrificed by cervical dislocation and the xenograft tumors were removed. Equal volumes of tumor tissue were then subcutaneously implanted into the rear flanks of 12 six-week-old nude mice. Following tumor formation (6 days), 5 mg/kg bromocriptine and/or fulvestrant was administered via intraperitoneal injection every three days for 24 days. The xenografts were monitored using calipers once every 3 days until the 30^th^ day, and the tumor volumes were calculated according to the following equation: V (mm^3^) = (length × width^2^)/2. On day 30 post-implantation, all mice were euthanized using exsanguination immediately after terminal CO_2_ administration, and the xenografts were carefully dissected. The tumors were weighed and processed in two ways; a proportion of each sample was processed for western blot analysis, and another was fixed for hematoxylin and eosin staining and immunohistochemical analysis.

### Statistical analysis

Statistical analyses were performed using SPSS 17.0 software (SPSS, Inc.). For patient samples, protein expression levels are presented as the mean ± SD. For *in vitro* and *in vivo* studies, values are expressed as the mean ± SEM. Two-tailed, independent Student's t-tests were used to compare continuous variables between two groups. Duncan's test was used to assess differences between multiple groups by using one-way ANOVA, and *P*<0.05 was considered to indicate a statistically significant difference.

## Results

### High ERα and PRLR, but low D2R expression in bromocriptine-resistant human pituitary adenomas and MMQ cells

**Figure 1:**
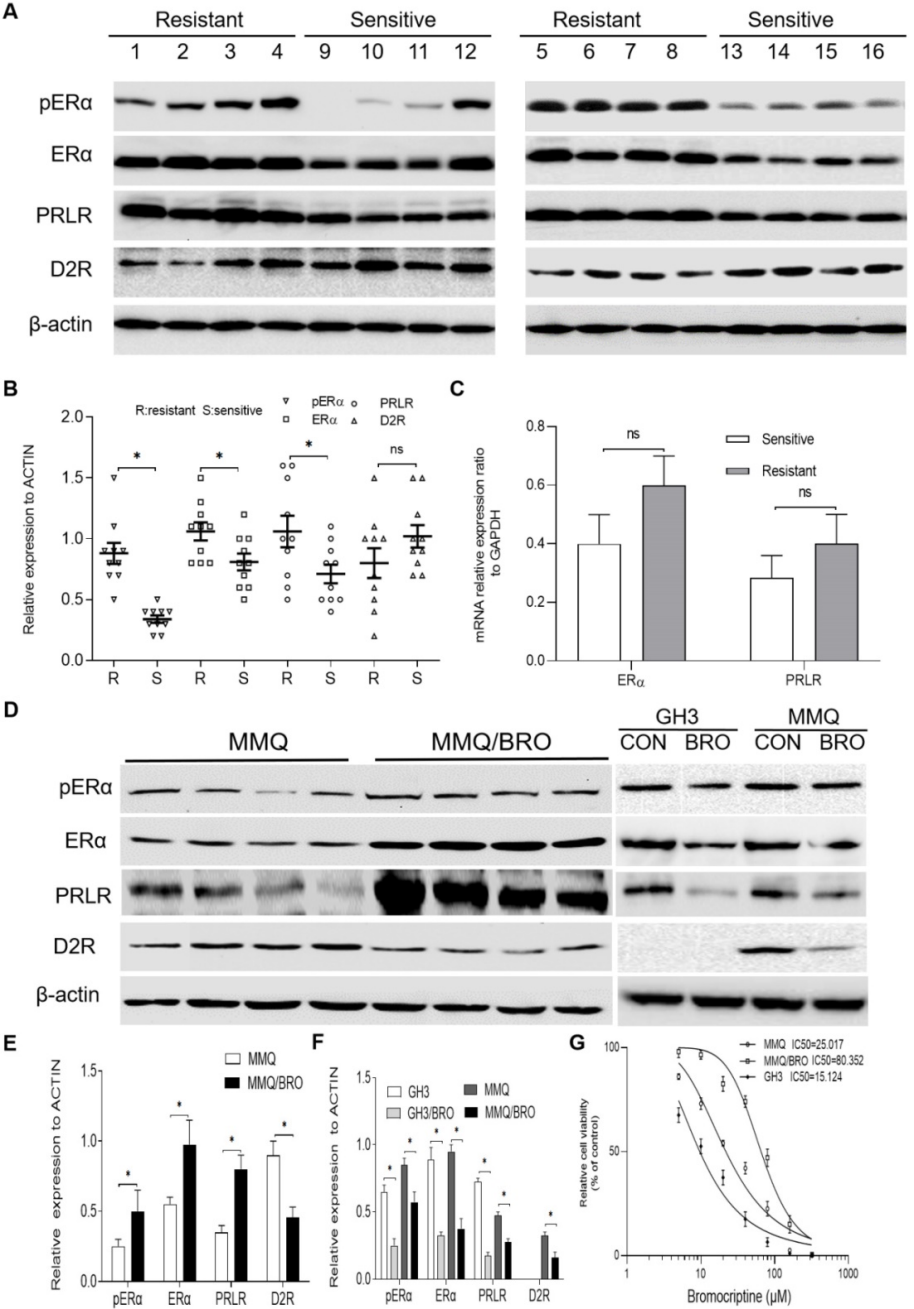
** Increased pERα, ERα and PRLR, but decreased D2R expression in bromocriptine‐resistant prolactinoma tissues and MMQ cells.** (**A**) Western blot analysis of pERα, ERα, PRLR and D2R protein expression in prolactinomas from bromocriptine‐resistant (No.1‐8) and ‐sensitive (No.9-16) patients. (**B**) Scatter plot of pERα, ERα, PRLR and D2R protein levels in 8 bromocriptine-sensitive (Sensitive) and 8 bromocriptine-resistant (Resistant) prolactinoma tissues. Data are presented as the mean ± SD (Student's t-test; ^**^P<0.05). (**C**) Quantification of ERαand PRLR mRNA levels in bromocriptine‐resistant and ‐sensitive prolactinoma tissues. (**D**) Western blot analysis of pERα, ERα, PRLR and D2R protein levels in bromocriptine‐resistant and ‐sensitive MMQ cells. The effect of bromocriptine (10 µM for 24h) on the pERα, ERα, PRLR and D2R protein levels in GH3 and MMQ cells was also examined. (**E**) Quantification of pERα, ERα, PRLR and D2R protein levels in bromocriptine‐resistant and ‐sensitive MMQ cells. (Student's t-test; ^**^P<0.05). (**F**) Quantification of pERα, ERα, PRLR and D2R protein levels in GH3 and MMQ cells treated with bromocriptine (10 µM) for 24 h. (Student's t-test; ^**^P<0.05). (**G**) IC_50_ for bromocriptine in MMQ cells, GH3 cells and bromocriptine-resistant prolactinoma cells (MMQ/BRO) by Cell Counting Kit 8 analysis. ERα, estrogen receptor α; pERα, phosphorylated ERα; PRLR, prolactin receptor; D2R, dopamine D2 receptor; BRO, bromocriptine; R, resistant; S, sensitive.

Previous studies have indicated that ER phosphorylation of serine residues in the AF-1 domain enhances ER-mediated transcription, and that cross-talk between phosphorylated ERα and other growth factor-signaling networks promotes therapy resistance 25. Since ERα is phosphorylated at Ser118 after binding estradiol or PRL 26,27, ERα phosphorylation and PRLR/D2R expression were analyzed in 8 bromocriptine-resistant and 8 bromocriptine-sensitive prolactinoma specimens using immunohistochemistry. Bromocriptine resistance has been commonly reported to accompany decreased levels of D2R 4-6,23,28,29. Moreover, antagonizing estrogen and dopamine can regulate the endocrine functions of the other, and estrogen may decrease D2R expression or attenuate functional coupling with its downstream effector 17,30,31. Based on these findings, D2R was also investigated in the present study. In line with previous studies 4,28,32,33, ERα, pERα and PRLR expression was significantly higher in bromocriptine-resistant than -sensitive samples. Drug-resistant samples also possessed lower D2R expression levels (Fig. 1A and B), though these results did not reach statistical significance. We further analyzed the mRNA expression of ERα and PRLR in these tissues by qPCR (Fig. 1C). A similar trend was observed in ERα and PRLR mRNA expression between bromocriptine-resistant and -sensitive samples tissues. However, this trend did not reach statistical significance.

In order to investigate the mechanism of bromocriptine resistance, a bromocriptine-resistant MMQ cell line (MMQ/BRO) was established following 6 months of treatment with increasing concentrations of bromocriptine. The CCK-8 assay results indicate that the MMQ/BRO cells IC_50_ value was ~80 μM, more than three times higher than that of MMQ cells (~25 μM).Compared with the parental MMQ cell line, MMQ/BRO cells possessed increased survival rates after following bromocriptine exposure (25 μM; Fig. 1F). As predicted, MMQ/BRO cells also possessed increased protein expression levels of pERα, ERα and PRLR, but decreased D2R expression (Fig. 1D and E). We also examined the effect of bromocriptine on the proliferation, pERα, ERα and PRLR protein levels in MMQ cells and GH3 cells. CCK8 cell proliferation assay showed that bromocriptine was more sensitive to GH3 cells than MMQ cells. The GH3 cells IC50 value was ~15 μM. Western blot showed that MMQ cells were ERα, PRLR and D2R protein postive cells, while GH3 cells were ERα and PRLR postive cells. There was no D2R expression in GH3 cells. After 10 μM bromocriptine treatment for 24 h, decreased PRLR and ERα protein expression levels were observed in MMQ and GH3 cells. These results suggest that bromocriptine treatment could alter the expression of ERα and PRLR, and PRLR and ERα signaling may be associated with bromocriptine resistance.

### Synergistic effects of PRL and E2 on cellular proliferation and D2R expression in prolactinoma cells

**Figure 2:**
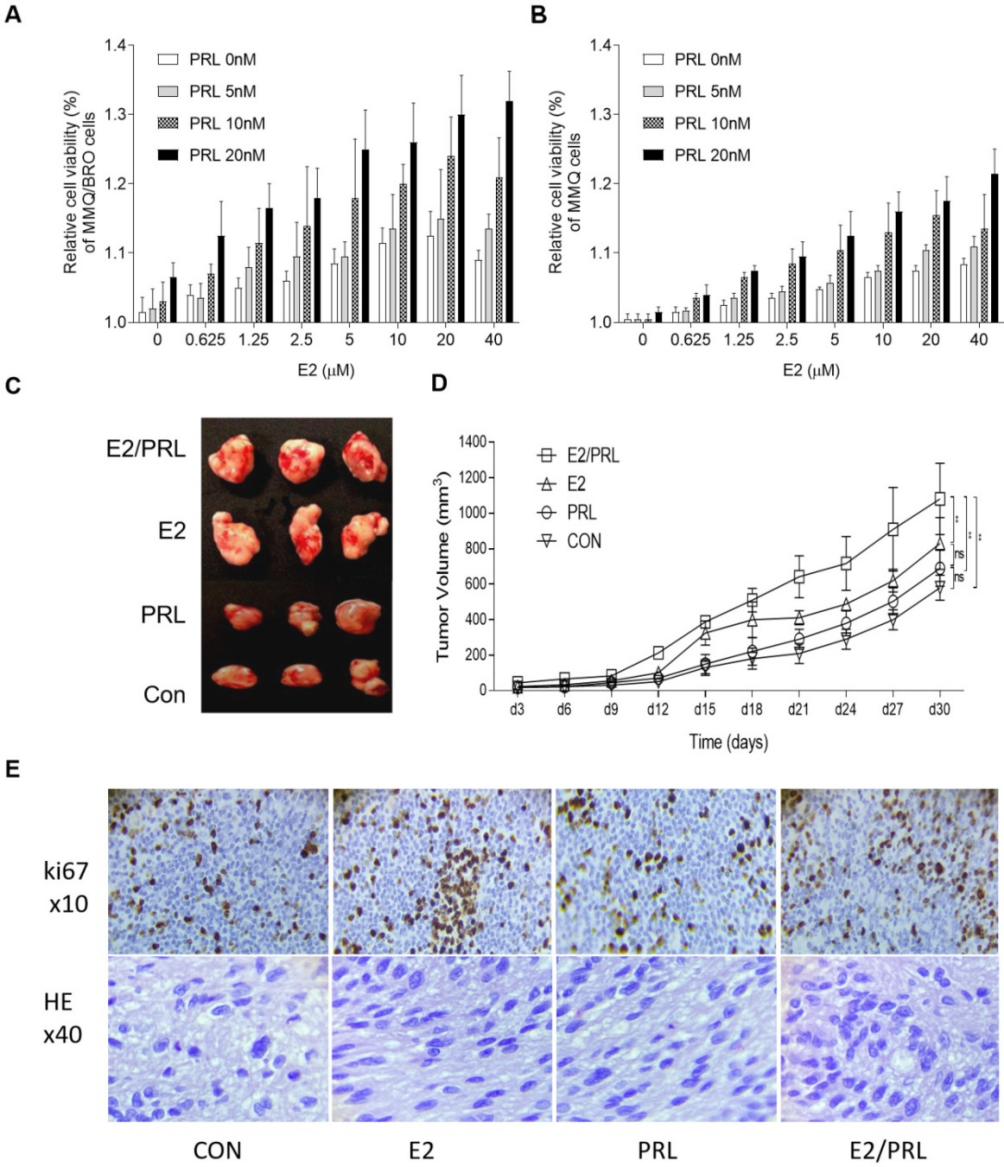
** Synergistic effects of PRL and E2 on the proliferation of MMQ/BRO cells and tumor growth in nude mice.** (**A**) MMQ/BRO cells and (**B**) MMQ cells were treated with PRL (0, 5, 10 and 20 ng/ml) in the presence or absence of E2 (0, 0.625, 1.25, 2.5, 5, 10, 20 and 40 µM) for three days. Cell viability was assessed using the Cell Counting Kit 8 assay. (**C**) Representative image of xenograft tumors isolated from nude mice. Human prolactinoma tissue xenograft mice were treated with E2 (25 ng/mouse), rhPRL (10 µg/mouse) or E2 plus rhPRL every three days for a total of 10 injections (n=4 per group). (**D**) Tumor volume as calculated according to the formula 0.5 x length x width^2^. (**E**) HE (magnification, x40) and Ki67 (magnification, x10) staining of human prolactinoma tissue xenograft tumors in nude mice (from left to right). All data are presented as the mean ± SD. ^**^P<0.05. PRL, prolactin; E2, estradiol; CON, control; rh, recombinant human; ns, not significant; HE, hematoxylin and eosin.

Since both estrogen and PRL serve key roles in the progression of pituitary adenomas, and as different hormones can interact with each other in various tissue types 26,34,35, it was hypothesized that estrogen and PRL may interact and exert synergistic biological effects in the tumor microenvironment. As HPA cells cannot not be completely isolated from human prolactinoma tissues, a small number of fibroblasts remain after long term coculture, which may decrease the reliability of proliferation and signaling results. In the present study, we used ERα+/PRLR+ /D2R+ MMQ cells but not ERα+/PRLR+/D2R- GH3 cells *in vitro* experiments, because ERα, PRLR and D2R protein was postive in human prolactinoma tissues. The proliferative capacity of MMQ/BRO and MMQ cells was determined. Serum-starved MMQ/BRO and MMQ cells were incubated with PRL (0, 5, 10 and 20 ng/ml) for three days in the presence or absence of E2 (0, 0.625, 1.25, 2.5, 5, 10, 20 and 40 μM). The use of PRL or E2 alone slightly enhanced cellular proliferation, and the proliferation was further increased following combination treatment (Fig. 2A and B). In order to further emulate the pathological characteristics of human prolactinomas, 200-μl prolactinoma tissue suspensions were injected into nude mice to establish a human prolactinoma xenograft model. The xenograft mice were then administered E2 (25 ng/mouse), recombinant human (rh)PRL (10 μg/mouse) or E2 plus rhPRL every three days for a total of 10 injections (n=3 per group). As predicted, xenografts in the PRL/E2 combination group were significantly larger and with a higher degree of ki67 staining than those in the PRL or E2 monotherapy groups (Fig. 2C-E). This observation confirms the synergistic proliferative effects of PRL and E2 on prolactinoma cells.

### PRL induces ERα activity, while E2 activates PRLR signaling

**Figure 3:**
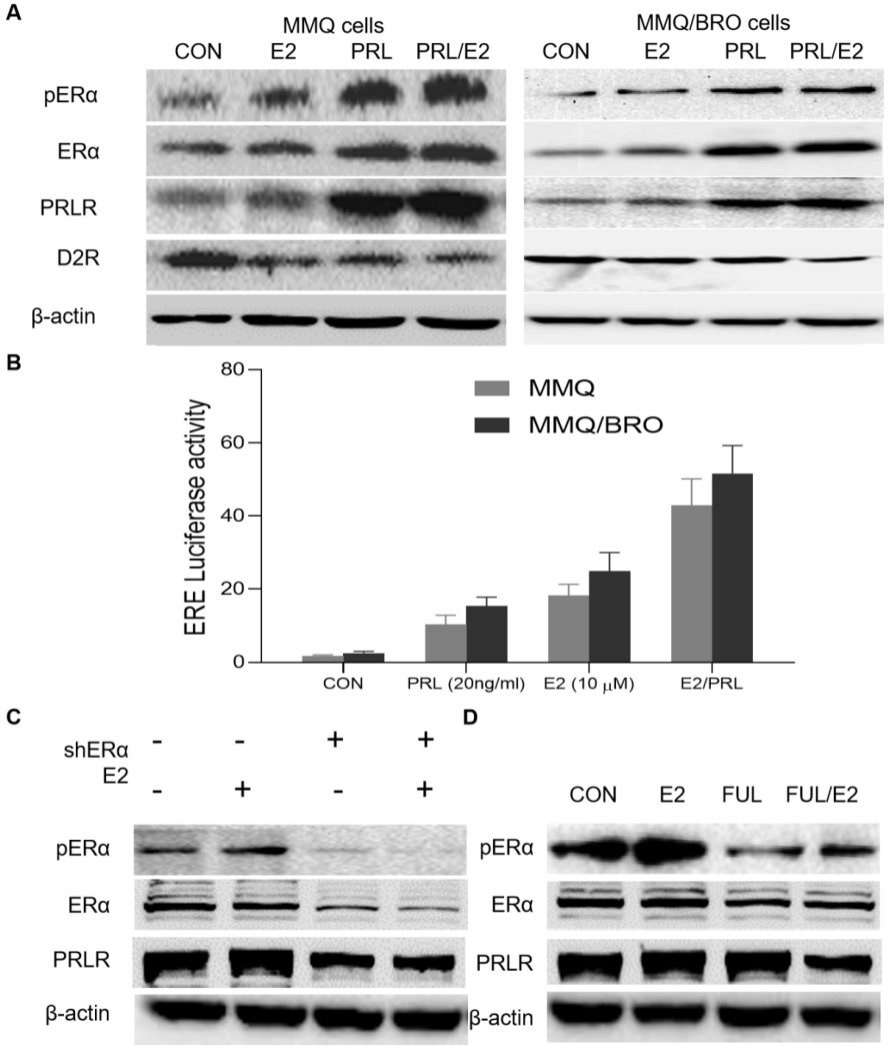
** PRL induces ERα activity, while E2 activates PRLR signaling.** (**A**) Effects of E2, PRL or E2 plus PRL on pERα, ERα, PRLR and D2R protein levels in MMQ and MMQ/BRO cells. MMQ or MMQ/BRO cells were serum-starved for 24 h, and then cultured with E2 (10 µM) or PRL (20 ng/ml) for 4 h. E2 and PRL stimulation resulted in upregulated pERα and PRLR, but decreased D2R expression. ERα had no notable effects. (**B**) Luciferase reporter assay demonstrating the synergistic effects of E2 and PRL on ER-mediated transcription. MMQ and MMQ/BRO cells were transfected with ERE (estrogen Response element) -Luc, and then treated with PRL (20 ng/ml) in the absence and presence of E2 (10 µM) for 4 h (Student's t-test; ^**^P<0.05). (**C**) Protein expression levels of pERα, ERα and PRLR in primary cultured HPA cells transiently transfected with control or ERα-specific shRNA (shERα), as detected by western blotting. ERα-knockdown (10 µM, 4 h) abolished E2-induced PRLR upregulation in HPA cells. (**D**) Fulvestrant abolished E2-induced pERα and PRLR upregulation in HPA cells. Primary cultured HPA cells were treated with E2 (10 µM) in the absence and presence of fulvestrant (20 nM) for 24 h. The protein expression levels of pERα, ERα and PRLR were detected by western blotting. E2, estradiol; PRL, prolactin; ERα, estrogen receptor α; pERα, phosphorylated ERα; PRLR, prolactin receptor; D2R, dopamine D2 receptor; CON, control; FUL, fulvestrant; sh, short hairpin (RNA); HPA, human pituitary adenoma.

To understand the mechanisms underlying the synergistic effects of PRL and E2 on prolactinoma cell proliferation, their influence on the expression of their canonical cognate receptor ERα and PRLR signaling markers was determined. In the present study, MMQ and MMQ/BRO cells transfected with the ERE-luc reporter were serum-starved for 24 h, and then cultured with E2 (10 μM) or PRL (20 ng/ml) for a further 4 h. E2 stimulation (10 μM, 4 h) resulted in upregulated pERα and PRLR, as well as decreased D2R expression. Additionally, PRL upregulation increased pERα and PRLR expression, while decreasing D2R expression. Interestingly, ERα expression was not obviously altered (Fig. 3A). The luciferase reporter assay indicated that both E2 and PRL induced ERE (estrogen response element) activity, but that a more profound effect was observed in the E2/PRL combination group (Fig. 3B). The results also indicate that E2 activates PRLR signaling, while PRL induces ERα phosphorylation and activity, suggesting a positive bilateral loop between the PRL/PRLR and E2/ ERα signaling pathways.

### E2 promotes PRLR activation via ERα phosphorylation

As aforementioned, E2 can upregulate PRLR expression, though the underlying mechanisms remain to be clarified. ER protein reportedly modifies gene transcription via protein-protein interactions 8,26, functioning as a coactivator protein by binding to other transcription factors 25. Previous studies have also shown that pERα associates with Sp1/C/EBPβ dimers in a DNA-independent manner, stimulating PRLR upregulation at the hPIII PRLR promoter 26,36-38. In order to obtain an appropriate human preclinical model for prolactinoma investigation, HPA cells were transiently transfected with pPLK/GFP+Puro-ERα shRNA. ERα-knockdown abolished E2-induced PRLR upregulation in HPA cells (Fig. 3C). Furthermore, addition of the ERα inhibitor fulvestrant (20 nM) for 24 h attenuated E2-induced PRLR upregulation in MMQ cells (Fig. 3D). These results demonstrate that pERα serves a vital role in the E2-induced upregulation of PRLR.

### PRL exposure induces ERα phosphorylation via the JAK2-PI3K/AKT-MEK/ERK signaling pathway

**Figure 4:**
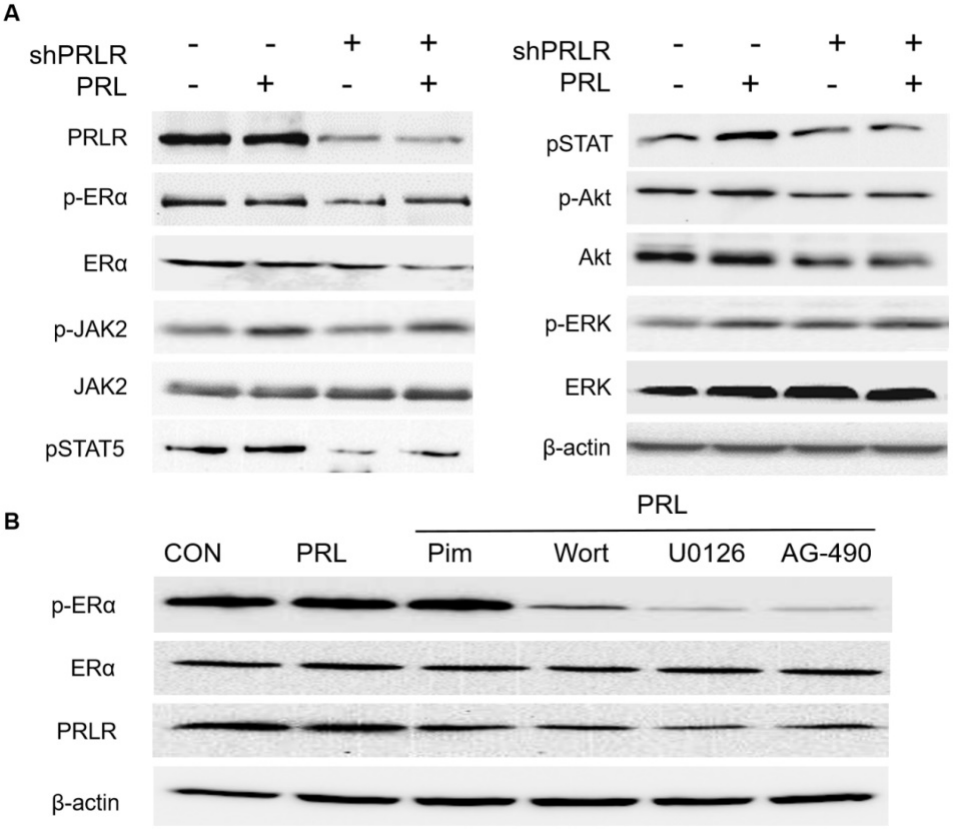
** PRL exposure induces ERα phosphorylation via the JAK2-PI3K/Akt-MEK/ERK pathway in primary cultured HPA cells.** (**A**) PRLR-knockdown abolishes PRL-induced JAK2-PI3K/Akt-MEK/ERK signaling. After overnight serum-starvation, HPA cells transiently transfected with control or PRLR-specific shRNA (shPRLR) were stimulated with PRL (20 ng/ml) for 4 h and the cells were harvested for western blotting using the indicated antibodies. (**B**) MMQ cells were treated with PRL (20 ng/ml) in the absence and presence of the JAK2 inhibitor AG-490 (50 μM), the PI3K inhibitor wortmannin (0.5 µM), the MEK inhibitor U0126 (10 µM) or the STAT5 inhibitor pimozide (5 µM). AG-490, wortmannin and U0126 partially blocked PRL-induced ERα phosphorylation, with the most profound effect in the AG-490 group; pimozide had no effect on PRL-induced ERα phosphorylation. PRL, prolactin; ERα, estrogen receptor α; HPA, human pituitary adenoma; PRLR, prolactin receptor; sh, short hairpin (RNA); CON. Control; Pim, pimozide; Wort, wortmannin.

PRL has been shown to promote the proliferation and differentiation of various types of cancer cells 16,25. It does so by activating associated signaling pathways via JAK2 phosphorylation of signal transducers and activators of transcription (STAT), protein kinase C and phosphatidylinositol 3-kinase (PI3K) and mitogen-activated protein kinase (MAPK) in cells that express PRLR 16,39-41. Although the majority of prolactinomas express both PRLR and ERα 13,16,29,35, to the best of our knowledge, the effects of PRL on prolactinoma cell ERα signaling are yet to be elucidated. Thus in the present study, HPA cells were starved of serum and estrogen for 24 h, and then treated with 20 ng/ml PRL for 4 h. Western blot analysis revealed a significant upregulation in pERα, pJAK2, pAKT, pERK, pSTAT5 and PRLR expression following PRL administration. Furthermore, shRNA PRLR-knockdown in HPA cells abolished the PRL-induced increase in pERα, pJAK2, pAKT, pERK and pSTAT5 (Fig. 4A). Since the primary functions of PRL are exerted via the JAK2/STAT5, PI3K/AKT and MAPK/ERK pathways, estrogen-starved MMQ cells were also treated with a specific inhibitor to determine the role of these signaling pathways in PRL-induced ERα phosphorylation. After 4 h, PRL was found to induce JAK2, AKT, ERK, pSTAT5 and ERα phosphorylation in MMQ cells. Preculturing cells with a JAK2 inhibitor (AG-490; 50 µM), a PI3K inhibitor (wortmannin; 0.5 µM) or a MEK inhibitor (U0126; 10 µM) partially blocked PRL-induced ERα phosphorylation; the most profound effect was observed in the AG-490 group, whereas 5 µM pimozide (a STAT5 inhibitor) had no effect on PRL-induced ERα phosphorylation (Fig. 4B). As JAK2 is known to activate PI3K and MEK signaling 25, these data indicate that the JAK2-PI3K/Akt-MEK/ERK pathway is involved in PRL-induced ERα phosphorylation, and that PRLR is required for PRL functionality.

### ERα-knockdown induces bromocriptine-mediated inhibition of proliferation and cancer cell stemness in pituitary adenoma cells

**Figure 5:**
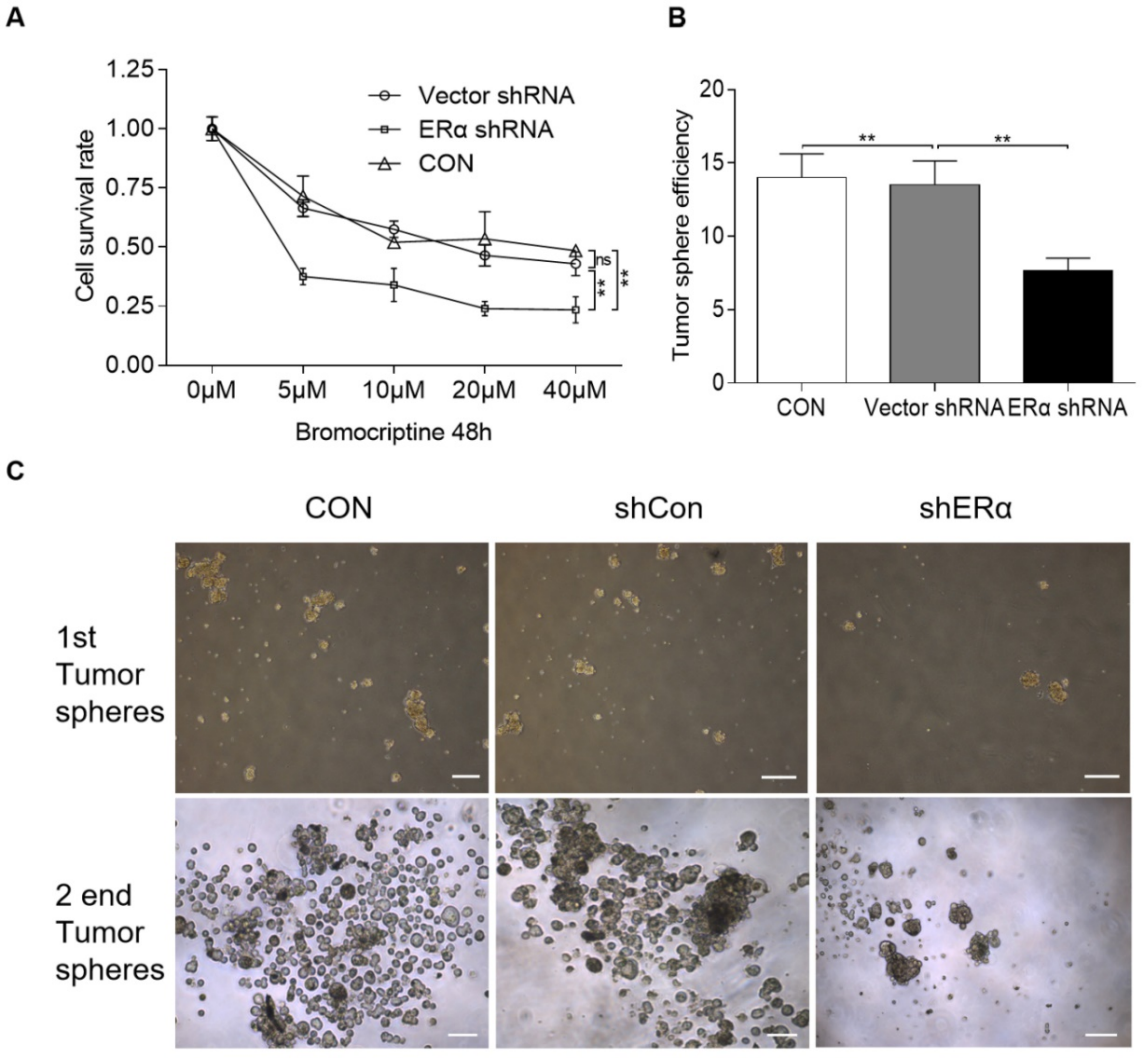
** ERα-knockdown significantly sensitizes cells to bromocriptine in HPA cells.** (**A**) Cell survival rate of shRNA-ERα or Vector-transfected HPA cells after treatment with different concentrations of bromocriptine (0, 5, 10, 20 and 40 µM) in the presence of E2 (10 µM) plus PRL (20 ng/ml) for three days (Student's t-test; ^**^P<0.05). (**B**) CD133+/nestin+ HPA stem-like cells were transiently transfected with control or ERα-specific shRNA (shERα) and cultured with bromocriptine (25 µM) for five days. Primary tumorspheres were counted and images were captured after five days. The average number of tumorsphere was recorded as the mean ± S.E. (^**^P≤ 0.05 vs. corresponding control cells). (**C**) Representative images of the tumor spheres. Sphere forming ability of HPA cells with ERα knockdown was significantly reduced, compared with that of vector and control group. sh, short hairpin (RNA); ERα, estrogen receptor α; HPA, human pituitary adenoma; E2, estradiol; PRL, prolactin; CON, control.

In our clinic, ~30% of prolactinomas are resistant to bromocriptine. The aforementioned results indicate that estrogen upregulates PRLR expression via pERα, while in turn, PRL induces ERα phosphorylation, resulting in a positive crosstalk loop. It was therefore hypothesized that ERα inhibition may sensitize resistant pituitary adenoma cells to bromocriptine treatment. The impact of ERα-knockdown on bromocriptine efficacy was investigated in HPA cells treated with 10 μM E2 and 20 ng/ml PRL. The cells were transiently transfected with pPLK/GFP+Puro-ERα or control shRNA, and then cultured with bromocriptine (0, 5, 10, 20 and 40 μM) for two days. The CCK-8 assay results indicate that ERα-knockdown significantly sensitizes cells to bromocriptine (Fig. 5A).

In a previous study, primary pituitary adenoma and stem-like cells were isolated using CD133 and Nestin magnetic beads 20,28. CD133+/nestin+ HPA cells form spheres and differentiate, suggesting the differentiation capacity and high tumorigenic ability of CD133+/nestin+ pituitary adenoma stem-like cells 20. The same protocol was used in the present study, where CD133+/nestin+ pituitary adenoma stem-like cells were isolated and transiently transfected with pPLK/GFP+Puro-ERα or the corresponding control shRNA. Single cells were then cultured with 25 μM bromocriptine and the average number of resultant colonies was recorded. Primary tumorspheres were counted and images were captured after 5 days. ERα shRNA-knockdown was found to exacerbate the bromocriptine-induced inhibition of tumorsphere formation in CD133+/nestin+ pituitary adenoma stem-like cells (Fig. 5B and C).

### ERα inhibition sensitizes bromocriptine-resistant pituitary adenoma cells *in vitro* and *in vivo*

**Figure 6:**
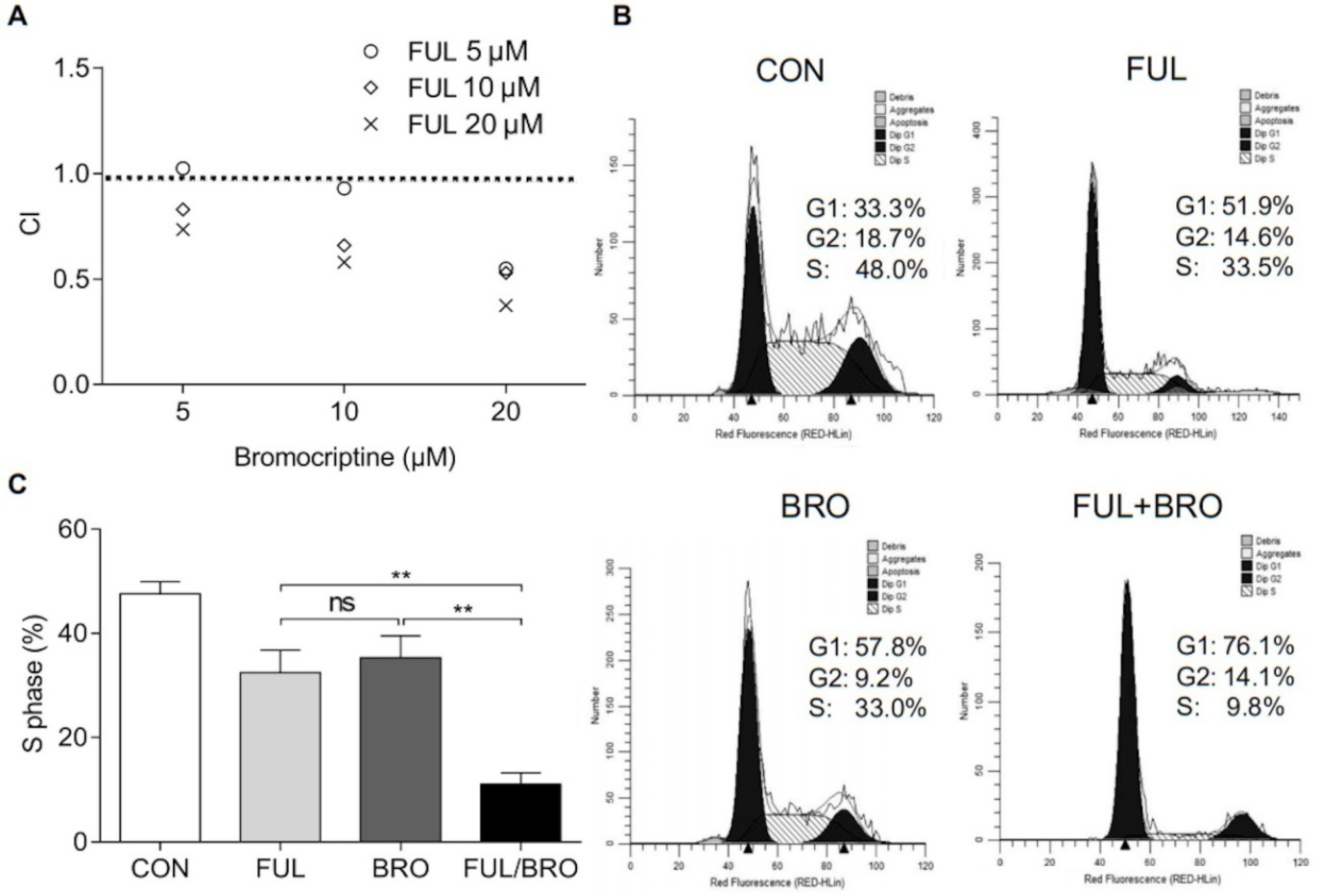
** Fulvestrant acts synergistically with bromocriptine in MMQ/BRO cells.** (**A**) CI of bromocriptine and fulvestrant, as determined by CCK-8 assay. Fulvestrant, an ERα inhibitor, synergistically enhanced bromocriptine-induced proliferative inhibition. MMQ/BRO cells were treated with bromocriptine (0, 5, 10 and 20 µM) in the absence or presence of fulvestrant ( 5, 10 and 20 nM) for 72 h, followed by CCK-8 assays. CI was calculated using CompuSyn software. Dose combinations with the CI<1.0 are considered synergistic. (**B and C**) Cell cycle analysis of MMQ/BRO cells treated with bromocriptine (25 µM) and/or fulvestrant (20 nM) for 24 h. Bromocriptine or fulvestrant monotherapy induced G_0_/G_1_ arrest and decreased the number of cells in S phase, whereas bromocriptine-induced growth inhibition was significantly sensitized in bromocriptine/fulvestrant co-treated cells, as indicated by a lower percentage of cells in S phase (Student's t-test; ^**^P<0.05). CI, combination index; CCK-8, Cell Counting Kit 8; ERα, estrogen receptor α; FUL, fulvestrant; CON, control; BRO, bromocriptine.

The effects of an ERα inhibitor on the proliferation of bromocriptine-resistant pituitary adenoma cells were further investigated under bromocriptine exposure. To determine whether fulvestrant acts synergistically with bromocriptine, MMQ/BRO cells were co-cultured with bromocriptine (5, 10 or 20 μM) and fulvestrant (5, 10 or 20 nM) for 72 h, and the combination indexes (CI) of bromocriptine and fulvestrant were analyzed using CompuSyn software. Fulvestrant synergistically enhanced bromocriptine-induced proliferative inhibition (as indicated by CI<1.0; Fig. 6A). Moreover, MMQ/BRO cell cycle analysis following 24 h bromocriptine and/or fulvestrant treatment revealed that when used alone, both bromocriptine and fulvestrant induced G_0_/G_1_ arrest and reduced the number of cells in the S phase. However, bromocriptine-induced inhibition was significantly increased in cells co-treated with fulvestrant, as indicated by a lower percentage of cells in S phase (Fig. 6B and C). These results validate ERα as a target for the sensitization of prolactinoma cells to bromocriptine.

**Figure 7:**
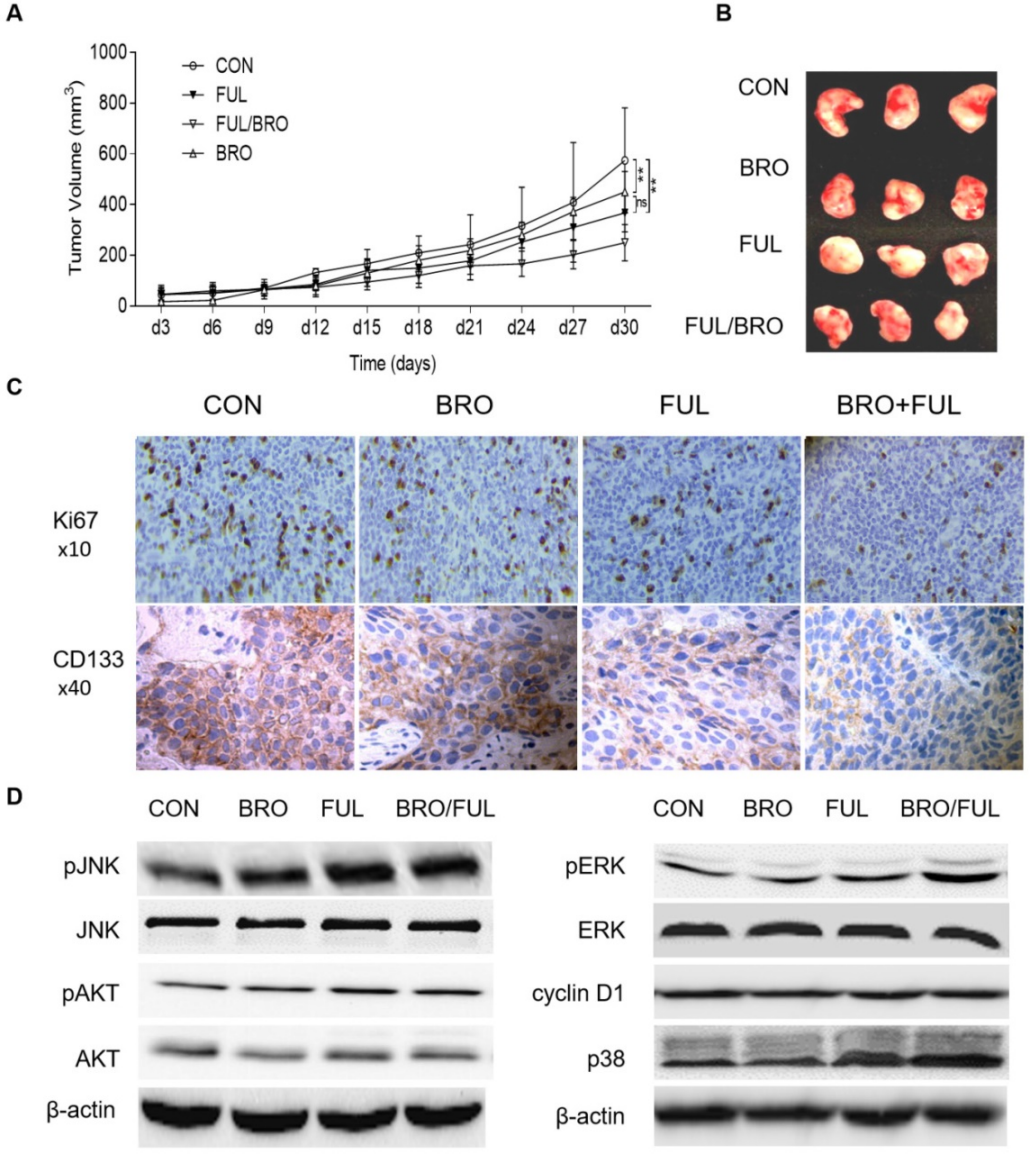
** Synergistic inhibitory effect of bromocriptine and fulvestrant on tumor growth in nude mice.** Human prolactinoma tissue xenograft mice were treated with bromocriptine (5 mg/kg) in the absence or presence of fulvestrant (5 mg/kg) every three days for a total of 8 injections (n=3 per group). (**A and B**) Tumor volumes as calculated according to the formula 0.5 x length x width^2^. All data are presented as the mean ± SD; ^**^P<0.05. (**C**) Representative immunohistochemistry images of CD133 and Ki67 staining in human prolactinoma tissue xenograft tumors. Lower CD133 and Ki67 protein expression levels in the BRO/FUL group. Scale bar represents 100 µm. (**D**) MMQ/BRO cells were co-cultured with bromocriptine (25 µM) and fulvestrant (20 nM) for 72 h followed by western blot analysis of the indicated markers. Combination of bromocriptine and fulvestrant induced activation of JNK-MEK/ERK-p38 MAPK signaling and cyclin D1 downregulation in MMQ/BRO cells. Data are based on three sets of experiments. CON, control; FUL, fulvestrant; BRO, bromocriptine.

*In vivo*, nude mice were injected with human prolactinoma tissue suspension. Following xenografts formation, bromocriptine (5 mg/kg) and fulvestrant (5 mg/kg) were administrated every three days in combination or alone. Compared with bromocriptine or fulvestrant alone, bromocriptine plus fulvestrant exerted a more profound antitumor effect on xenograft growth (Fig. 7A and B). Moreover, xenograft tissues treated with bromocriptine plus fulvestrant possessed lower Ki67 and CD133 expression levels (Fig. 7C). These data suggest that ERα inhibition enhances the antitumor proliferative and stemness activities of bromocriptine both *in vitro* and *in vivo.*

### JNK-MEK/ERK-p38 and cyclin D1 signaling in fulvestrant-induced bromocriptine sensitization

To further investigate the synergistic effects of fulvestrant and bromocriptine on downstream signaling molecules, ERα- and bromocriptine-induced signaling was observed in MMQ cells treated with bromocriptine and fulvestrant alone or in combination. Key downstream markers of ERα- or bromocriptine-induced signaling, such as p38, AKT, ERK, JNK and cyclin D1 9,17,30,42-44, were investigated by western blotting following co-culture with bromocriptine (25 μM) and fulvestrant (20 nM) for 72 h. Enhanced pJNK1/2, pERK1/2 and p38, but decreased cyclin D1 expression was observed in the combination group, while pAKT was not obviously altered (Fig. 7D). These results suggest that fulvestrant-induced activation of JNK-MEK/ERK-p38 MAPK signaling and cyclin D1 downregulation serves a role in bromocriptine-induced sensitization following ERα/PRLR crosstalk blockage. JNK-MEK/ERK-p38 MAPK- and cyclin D1-associated cell cycle arrest may also be involved in fulvestrant-induced bromocriptine sensitization.

## Discussion

The present study aimed to investigate the effects of ERα and PRLR signaling crosstalk on bromocriptine resistance in prolactinoma cells. High ERα and PRL, but low D2R expression was found to be associated with bromocriptine resistance in prolactinomas. Furthermore, estrogen was found to upregulate PRLR expression via pERα, while PRL induced ERα phosphorylation, forming a positive feedback loop. Blocking this loop via ERα inhibition resulted in downregulated PRLR/ERα signaling, exacerbating bromocriptine-induced cell cycle arrest, antitumor stemness and anti-proliferative activity. Specifically, ERα-knockdown significantly sensitized these cells to bromocriptine, and the combination of bromocriptine and the ERα inhibitor fulvestrant resulted in significantly enhanced retardation of bromocriptine-resistant cells, compared with either bromocriptine or fulvestrant alone. The results of the present study identified ERα/PRLR crosstalk as a mediator of bromocriptine responsiveness in prolactinomas, and demonstrated that blocking ERα/PRLR crosstalk exacerbates JNK-MEK/ERK-p38 and cyclin D1 downregulation, which further restores bromocriptine sensitivity in prolactinomas. These findings have important implications on the future use of bromocriptine as an anti-prolactinoma agent, for which ERα may be used as an adjuvant sensitizing therapeutic target.

In the present study, bromocriptine-resistant prolactinomas displayed high pERα, ERα and PRLR, but low D2R expression levels, which is of great significance in exploring the influencing factors of bromocriptine efficacy. PRL activity is primarily mediated via PRLR 40,41,45, while the estrogen receptors, especially ERα, mediate the majority of the biological effects of estrogen 9,11,36-38,46,47. To date, the expression levels of ERα in pituitary adenoma tissues remain to be clarified. Carretero *et al* 14 observed that all prolactinomas stained positively for ERα. In terms of bromocriptine resistance, Zhang *et al* 5 found that tumor volume was positively correlated with PRL concentration and ERα expression in bromocriptine-resistant prolactinomas 5. Moreover, Delgrange *et al* 41 also reported that prolactinomas possess lower ERα expression levels in men, and that ERα expression is associated with a higher tumor grade, resistance to treatment and a poorer prognosis. Besides ERα, previous studies have indicated that most prolactinomas express PRLR 17, and that PRL increases estrogen responsiveness in multiple tissue types 41. Moreover, an increased circulating PRL level is linked to the risk of ER-positive breast cancer 40. Since both the estrogen/ERα and PRL/PRLR signaling pathways are recognized as pathogenic mediators of pituitary adenoma, these aforementioned findings suggest that estrogen/ERα and PRL/PRLR signaling may be involved in the therapeutic resistance of prolactinomas.

In our clinic, bromocriptine is the first-line therapy choice. However, ~30% of patients still do not respond to bromocriptine therapy. Moreover, as a result of increased drug resistance, the potency of bromocriptine treatment can be reduced over time. Therefore, alternative and complementary therapies are urgently required. Previous studies have suggested that bromocriptine resistance is frequently accompanied by D2R downregulation 4,6,23,28. Many researcher inferred that 15-20% of the patients were resistant to the bromocriptine treatment due to decreased expression and/or signaling of D2R, because bromocriptine-resistant samples possessed lower D2R expression levels than bromocriptine sensitive ones 6,23. However, there was no evidence for a direct correlation between bromocriptine resistance and D2R downregulation. Because PRL regulates its own secretion by short-loop negative feedback on dopamine releasing neurons 16, D2R downregulation may simply be a consequence of negative feedback rather than a trigger for hyperprolactinemia in bromocriptine resistant prolactinomas. In this study, drug-resistant samples also possessed lower D2R expression levels then drug sensitive ones, which is in line with the previous reports and inference. However, in our study, D2R negative GH3 cells are more sensitive to bromocriptine than D2R positive MMQ cells. The mechanism by which bromocriptine suppresses the pituitary adenoma cells is more enigmatic. Given the close interactions and overlapping functions between members of the PRL/GH family, this controversial result should not be viewed in isolation, especially in humans. Because tumor cells grow on plastic culture dishes do not truly represent the behavior of tumors in humans. This controversial results may be due to the cross-talk between hormones, the different drug sensitivity among tumor cells, Or maybe it's simply because of the difference in the response of different cells to drugs, which should be undertaken in future research. In our study, Drug-resistant samples displayed lower D2R expression levels. However, these results did not reach statistical significance. These results may be due to the following reasons. The total amount of DR2 may be affected by a variety of pathophysiological factors *in vivo*, so there may be no statistical difference in the total amount. In addition, the overall sample size of this study is too small, and more patient samples are needed for further study. Moreover, other reports have indicated high levels of ERα expression in bromocriptine-resistant prolactinomas 5,22,48, suggesting that ERα may also be a critical factor for bromocriptine efficacy. In addition to ERα, high PRLR expression was also observed in bromocriptine-resistant prolactinomas in the present study. In line with PRL/PRLR and estrogen/ERα co-expression in the prolactinoma microenvironment, and the fact that estrogen may enhance inter-hormonal crosstalk, which in turn contributes to therapeutic resistance 19,34,49. These data highlight the necessity of further research into the contribution of ERα and PRLR crosstalk to bromocriptine resistance.

Due to the limitations of the clinical data, *in vitro* experimentation was conducted to determine the potential effects of estrogen or PRL on their canonical pathways, including ERα, pERα, PRLR and D2R expression in pituitary adenoma cells. MMQ/BRO cells were co-cultured with E2 or PRL, which revealed that E2 exposure can upregulate pERα and PRLR expression, while PRL in the absence of estrogen induces ERα phosphorylation, both of which downregulate D2R expression. Furthermore, compared with E2 or PRL treatment alone, estrogen/PRL co-treatment increased MMQ/BRO cell proliferation and prolactinoma tissue xenograft growth in nude mice. These data confirm the interaction between estrogen/ERα and PRL/PRLR signaling in tumor growth and the progression of prolactinomas. The study also demonstrated that estrogen upregulates PRLR expression via pERα and that PRL induces ERα phosphorylation, suggesting that PRLR may be an important regulator of estrogen responsiveness, and that pERα may be a key component in PRL-induced oncogenesis.

The present study indicated the interaction between the estrogen/ERα and PRL/PRLR signaling pathways, though the precise molecular mechanisms are not completely understood. Estrogen primarily exerts its biological effects through membrane-localized ERα. E2/ERα has been reported to combine with C/EBPβ/SP1 and induce transcriptional activation of the generic hPIII promoter, activating PRLR expression in MCF-7 breast cancer cells 25. Evidence also suggests that PRL activates the JAK2-PI3K/Akt-MEK/ERK pathway by binding to PRLR 18,39-41, and thus acting as a survival agent and mitogen. Previous studies have also indicated that PRLR antagonists induce human breast cancer cell death *in vitro* and abrogate tumorigenesis *in vivo*, while PRLR-knockout in mice prevents mammary tumorigenesis 16,18,40,41, which demonstrates that PRL is essential for the proliferation and survival of these cells. In the current study, both estrogen and PRL promoted ERα phosphorylation. Estrogen can induce ERα phosphorylation and upregulate PRLR via pERα signaling, while PRL induces ERα phosphorylation via the JAK2-PI3K/Akt-MEK/ERK pathway. ERα inhibition not only abolished E2-induced PRLR upregulation, but also abrogated E2/PRL-induced ERα phosphorylation. Furthermore, ERα shRNA-knockdown exacerbated the bromocriptine-induced inhibition of cellular proliferation and tumorsphere formation in primary cultured HPA cells and their counterpart CD133+/nestin+ pituitary adenoma stem-like cells. A bromocriptine-resistant MMQ cell line was also constructed, and was used to determine that ERα inhibition can sensitize bromocriptine-resistant MMQ cells. Furthermore, bromocriptine/fulvestrant co-treatment resulted in G_0_/G_1_ arrest and decreased the number of S phase MMQ/BRO cells, as well as Ki67, ERα, PRLR and CD133 expression levels in human prolactinoma xenograft tissues. Our *in vitro* study showed that bromocriptine treatment could alter the expression of ERα and PRLR in MMQ cells. In human pituitary tumor tissues, we have only encountered one patient who first underwent craniotomy for large prolactinoma, and then, the patient received three month bromocriptine treatment for tumor residues in the sella. The patient underwent transnasal sphenoidal pituitary tumor resection due to the invalid of bromocriptine administration. We compared the pathological slices of the patient's first and second operations. We found that the expression of ERα and PRLR was decreased in the second surgery tumor tissues after oral administration of Bromocriptine. However, the significance of this founding is limited, because we found that the protein expression of the samples from the first and second surgeries in one patient may be different, even the samples taken from the same surgery may be different due to different sites. Large-scale clinical case support is needed for the effects of drugs on ERα and PRLR protein levels in pituitary tumor tissues.

Since the results of the present study showed that ERα and PRLR positively regulate each other to form a positive regulatory loop, which may promote tumor proliferation, stemness and ultimately bromocriptine resistance, understanding the downstream mediators of ERα/PRLR interactions is of great significance in elucidating the underlying mechanisms. To further clarify this issue, blocking this interaction with fulvestrant (an ERα inhibitor) in the bromocriptine group significantly enhanced p-JNK1/2, p-ERK1/2 and p38, but decreased cyclin D1 expression. Previous studies have also revealed that persistent activation of ERK signaling produces an anti-proliferative and anti-tumorigenic effect. In addition, both the p38 and JNK pathways promote apoptosis, a cytotoxic cellular process. The current data suggest that fulvestrant induces bromocriptine sensitization by reducing proliferation, and induces apoptosis and cell cycle arrest by activating JNK-MEK/ERK-p38 MAPK signaling and downregulating cyclin D1.

To the best of our knowledge, the results of the present study demonstrate for the first time that ERα inhibition restores bromocriptine sensitivity, and that targeting ERα/PRLR crosstalk may counteract bromocriptine-resistance in prolactinomas. In clinical practice, patients with prolactinomas are administered bromocriptine as the first line of treatment. Once resistance occurs, fulvestrant alone is usually administrated as an alternative therapeutic strategy; however, the response is often unsatisfactory 6,7,23,27. Fulvestrant is a synthetic estrogen receptor antagonist, fulvestrant binds competitively to estrogen receptors and results in estrogen receptor deformation and decreased estrogen binding. The ERα inhibitor fulvestrant has been approved by the FDA and used in clinical practice for numerous years with few side effects; fulvestrant monotherapy shows little efficiency in prolactinoma treatment. Therefore, the results of the present study suggest that combining fulvestrant and bromocriptine may provide an additional means to counteract resistance in clinical practice, though this requires further investigation.

## Highlights

- High estrogen receptor α (ERα) and prolactin receptor (PRLR), but low dopamine D2 receptor (D2R) expression in bromocriptine-resistant human pituitary adenomas;
- Synergistic effects of prolactin and estradiol on cellular proliferation and D2R expression in prolactinoma cells;
- PRL induces ERα phosphorylation while estrogen promotes PRLR upregulation via pERα;
- ERα inhibition restores bromocriptine sensitivity in pituitary adenoma cells.
