# Estrogen receptor α/prolactin receptor bilateral crosstalk promotes bromocriptine resistance in prolactinomas: Erratum

**DOI:** 10.7150/ijms.71659

**Published:** 2022-05-03

**Authors:** Zhengzheng Xiao, Xiaoli Yang, Kun Zhang, Zebin Liu, Zheng Shao, Chaojun Song, Xiaobin Wang, Zhengwei Li

**Affiliations:** 1 Department of Henan Key Laboratory of Cancer Epigenetics; Cancer Institute, Department of Neurosurgery, The First Affiliated Hospital and College of Clinical Medicine of Henan University of Science and Technology, Luoyang, Henan 471003.; 2 Department of General Practice, The First Affiliated Hospital and College of Clinical Medicine of Henan University of Science and Technology, Luoyang, Henan 471003.; 3 Spine Tumor Center, Department of Orthopedic Oncology, Changzheng Hospital, Second Military Medical University, Shanghai 210011.; 4 Carson International Cancer Centre, Shenzhen University General Hospital and Shenzhen University Clinical Medical Academy Centre, Shenzhen University, Shenzhen, Guangdong 518000.; 5 Department of Neurosurgery, Zhongnan Hospital of Wuhan University, Wuhan, Hubei 430071, P.R. China.

The images of original Figure 2E and Figure 7C were incorrectly assembled. The figures should be corrected as follows. All authors were informed and approved the corrected figures.

## Figures and Tables

**Figure 2:**
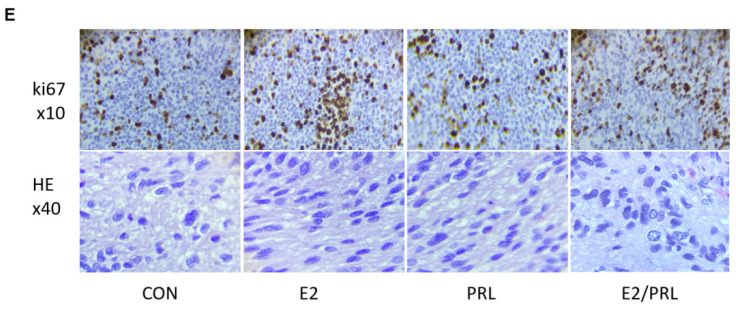
Synergistic effects of PRL and E2 on the proliferation of MMQ/BRO cells and tumor growth in nude mice. (E) HE (magnification, x40) and Ki67 (magnification, x10) staining of human prolactinoma tissue xenograft tumors in nude mice (from left to right).PRL, prolactin; E2, estradiol; CON, control; rh, recombinant human; ns, not significant; HE, hematoxylin and eosin.

**Figure 7:**
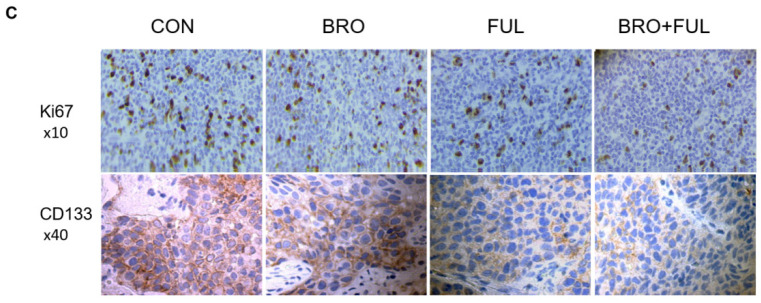
Synergistic inhibitory effect of bromocriptine and fulvestrant on tumor growth in nude mice. (**C**) Representative immunohistochemistry images of CD133 and Ki67 staining in human prolactinoma tissue xenograft tumors. Lower CD133 and Ki67 protein expression levels in the BRO/FUL group. Scale bar represents 100 µm. CON, control; FUL, fulvestrant; BRO, bromocriptine.

